# Supplementary material for: Empirical methods for controlling false positives and estimating confidence in ChIP-Seq peaks
Source: BMC Bioinformatics. 2008 Dec 5;9:523. doi: 10.1186/1471-2105-9-523 (PMC2628906; doi:10.1186/1471-2105-9-523)
Supplement: Additional File 1 — A variety of html documents from the USeq web site detailing the available applications, their best usage, output file type descriptions, command line menus, etc. [file 1471-2105-9-523-S1.zip › USeqUserGuides/JavaDoc.zip!/JavaDoc/util/bio/parsers/gff/GffParser.html]

GffParser


|  |  |  |
| --- | --- | --- |
| **Overview** \| **Package** \| **Class** \| **Use** \| **Tree** \| **Deprecated** \| **Index** \| **Help** | |  |
| **PREV CLASS**   **NEXT CLASS** | **FRAMES**    **NO FRAMES**     **All Classes** |  |
| SUMMARY: NESTED \| FIELD \| CONSTR \| METHOD | DETAIL: FIELD \| CONSTR \| METHOD |  |


---


## util.bio.parsers.gff Class GffParser

```
java.lang.Object
  util.bio.parsers.gff.GffParser
```

---

``` public class GffParser extends java.lang.Object ```

Parses GFF files extracting the annotation according to GFF(2).
http://www.sanger.ac.uk/Software/formats/GFF/GFF\_Spec.shtml
tabtabtabtabtabtab
tabtab[space delimited key=value; key=value; attributes]
Uses some hashes to count the sources, features, and attributes
Generates an array of GffFeature objects, see to learn about what's
used to parse the Attributes.
This parser needs to be tested on each new GFF file, the GFF format is so
flexible as to cause all kinds of problems.
Works with flybase, and the drosophila release 3.1 gff annotation.

---

**Constructor Summary**

`GffParser(java.io.File file,
int startNt,
int stopNt)`


| **Method Summary** | |
| --- | --- |
| `GffFeature[]` | `getGffFeatures()` |
| `void` | `quantifyItems()` |

**Methods inherited from class java.lang.Object**

`equals, getClass, hashCode, notify, notifyAll, toString, wait, wait, wait`

**Constructor Detail**

### GffParser

```
public GffParser(java.io.File file,
                 int startNt,
                 int stopNt)
```


**Method Detail**

### getGffFeatures

```
public GffFeature[] getGffFeatures()
```

---


### quantifyItems

```
public void quantifyItems()
```


---


|  |  |  |
| --- | --- | --- |
| **Overview** \| **Package** \| **Class** \| **Use** \| **Tree** \| **Deprecated** \| **Index** \| **Help** | |  |
| **PREV CLASS**   **NEXT CLASS** | **FRAMES**    **NO FRAMES**     **All Classes** |  |
| SUMMARY: NESTED \| FIELD \| CONSTR \| METHOD | DETAIL: FIELD \| CONSTR \| METHOD |  |


---
